# Hyaluronidase Promote Transdermal Diffusion of Small Sized Curcumin Nanocrystal by Dissolving Microneedles Delivery

**DOI:** 10.3390/pharmaceutics15030788

**Published:** 2023-02-27

**Authors:** Xiaoqing Miao, Jingru Zhao, Hong Xiang, Xiaoxi Shi

**Affiliations:** 1 Marine College, Shandong University, Weihai 264209, China; 2 SDU-ANU Joint Science College, Shandong University, Weihai 264209, China

**Keywords:** hyaluronidase, curcumin, nanocrystals, transdermal diffusion, microneedles

## Abstract

Hyaluronidase is clinically used in treating many skin diseases due to its good permeability-promoting effect, which may motivate the diffusion and absorption of drugs. To verify the penetration osmotic effect of hyaluronidase in microneedles, 55 nm-size curcumin nanocrystals were fabricated and loaded into microneedles containing hyaluronidase in the tip. Microneedles with bullet shape and backing layer of 20% PVA + 20% PVP K30 (*w*/*v*) showed excellent performance. The microneedles were able to pierce the skin effectively with a skin insert rate of 90% and demonstrated good mechanical strength. In the in vitro permeation assay, with the increase of hyaluronidase concentration at the tip of the needle, the cumulative release of curcumin increased, as well as the skin retention decreased. In addition, compared with the microneedles without hyaluronidase, the microneedles containing hyaluronidase in the tip exhibited a larger drug diffusion area and deeper diffusion depth. In conclusion, hyaluronidase could effectively promote the transdermal diffusion and absorption of the drug.

## 1. Introduction

Curcumin (CUR) has extensive physiological and pharmacological activities, such as antioxidant [1], anti-inflammatory [2], antihypertensive [3], antitumor [4,5] and so on. In addition, it is also clinically used in the treatment of multiple organ injuries [6], neurological diseases [7], cancer [8], depression [9] and other diseases. However, poor solubility, oral bioavailability (only 1% [10]) and intestinal permeability [11,12] limit further applications. To address the instability and insolubility problems, the pharmaceutical modification of CUR, such as CUR liposomes [13], gels [14], and biotextiles containing CUR [15], were proposed and applied in the treatment of breast cancer and psoriasis. The mechanism of CUR works on skin involved as follows [16]: when the medium evaporates, continuous aqueous menisci are generated between CUR particles and skin, triggering local swelling of the skin and achieving highly passive penetration of CUR. 

Compared with oral or injection, transdermal administration has many advantages, such as low pain and low dose [17]. Relevant drug preparations are applied to human skin, achieving local or systemic drug delivery. As the major impediment to transdermal drug delivery, skin is composed of four parts: stratum corneum (SC), epidermis, dermis, and hypodermis layers from top to bottom [18]. Only some small molecules and medium lipophilic molecules penetrate the SC barrier, while many insoluble drugs are difficult to pass through the skin [19]. Therefore, to promote the absorption of insoluble drugs, many strategies were applied: ① permeation enhancer (polyethylene glycol, polysaccharides, propylene glycol, alcohol, etc. [20,21,22,23,24]); ② pharmaceutical technologies (nanocrystals (NCs), liposomes, microemulsion formulations, etc.); ③ physical technologies (electroporation, iontophoresis, microneedles (MNs), etc.) [25].

Hyaluronic acid (HA), a glycosaminoglycan, is one of the main components of the skin extracellular matrix (ECM) [26]. Hyaluronidase (HAase) could reduce the activity of HA by cutting the glycosidic bond [27]. When HAase meets HA in the dermis, HA is depolymerized under the depolymerization action of HAase. Then the dermis tissue is loosened, and the ECM barrier is destroyed, resulting in enhancing a larger area of drug diffusion and deeper penetration distance [28]. Since the discovery of the biological activity of HAase, it was also clinically used in the treatment of chemotherapy extravasation [29], hypertrophic scar [30], local scleroderma, melanoma [31], and other diseases due to its optimistic effect on enhancing diffusion kinetics. In addition, combination therapy is also an important means of HAase application as well [32,33]. Co-loading HAase and hydrophilic metformin hydrochloride into MNs could significantly improve the efficiency of transcutaneous penetration [28]. However, as we know, most active pharmaceutical ingredients are poorly water soluble, and HAase loaded in MNs may increase the penetration of this kind of drug.

MN is a needle-like medical instrument, which is composed of many pretty thin and short, tiny needle tips arranged on the patch by matrix. MNs pass through the SC by the tip of the needle, leaving a nano-level channel aperture on the skin for the delivery of drugs [34]. Usually, the small needle tip only pierces the papillary layer without damaging the nerves and capillaries of the dermis [35]. Dissolving MNs could relieve the symptoms of needle phobia and avoid the pain caused by injections. The drug loading of NCs is higher than that of other nanoparticles, such as micelles and liposomes. Therefore, loading NCs into MNs could obtain a relatively high drug-loading capacity. [36] In addition, some insoluble drugs such as CUR [37] and albendazole [38] were successfully delivered by MNs to treat many skin diseases [39], such as skin cancer [40], acne, scars [41], psoriasis [42] and hair loss [43]. Previous MNs based studies focused on the advantages of overcoming the SC barrier to enhance drug delivery, while the effect of the ECM barrier on the skin penetration of poorly soluble drugs may be neglected [44]. Hence in this work, we focused on the HAase to depolymerize the HA and loosen the ECM barrier.

Therefore, in order to overcome the skin ECM barrier and achieve deep penetration of poorly soluble drugs, 55 nm-size CUR-NCs were prepared and loaded into dissolving MNs with or without HAase. The effect of HAase on enhancing the skin penetration of insoluble drugs was evaluated by in vitro percutaneous penetration and diffusion experiments.

## 2. Materials and Methods

### 2.1. Chemicals and Materials

CUR (purity > 98%) was purchased from Fuyumi Biological Technology Co., Ltd. (Nantong, China). HAase was purchased from Yante Biological Technology Co., Ltd. (Shanghai, China). Polyvinylpyrrolidone (PVP K30 and PVP K90) was purchased from Macklin (Shanghai, China). Polyethylene glycol 400 (PEG 400) was purchased from China National Pharmaceutical Group Co., Ltd. (Beijing, China). All other chemical reagents were analytical or chromatographic grade.

### 2.2. Preparation of 55 nm-Size CUR-NCs

First, 55 nm-size CUR-NCs were prepared by the method of stirred precipitation [12]. The organic phase solution was obtained by dissolving 10 mg CUR drug substance in 2 mL anhydrous ethanol. In the same way, 60 mg PVP K90 was dissolved in 6 mL deionized water to prepare the aqueous phase solution. Under stirring at 1000 rpm, 2 mL of CUR ethanol solution (5 mg/mL) was uniformly added to 18 mL of aqueous phase containing 0.33% PVP K90 (*w*/*v*) via a 1 mL syringe. The 30 kDa Amicon^®^ Ultra-15 centrifugal filtration device (Merck, Darmstadt, Germany) was used to remove the organic solvent. 

### 2.3. Characterization of CUR-NCs

The particle size and polydispersity index (PDI) of the CUR-NCs were measured by Nano^®^ Zetasizer (Malvern Instruments, Worcestershire, UK). The morphology of the CUR-NC was observed by Nova Nano SEM (FEI, Hillsboro, OR, USA).

### 2.4. Preparation of HAase-Loaded CUR-MNs

The CUR-MNs were fabricated by the method of double-layer casting. The concentrated CUR-NCs suspension of 1 mL was mixed with 1 mL of 60% PVP K30 (*w*/*v*) gel. PVP K30 (30%, *w*/*v*) solution was used as the solvent to dissolve HAase to form the enzyme solution. First, the enzyme solution was filled into the polydimethylsiloxane (PDMS) molds (① 15 × 15 quadrilateral cone, height 600 µm, radius 290 µm; ② 15 × 15 cone, height 600 µm, diameter 280 µm; ③ 15 × 15 bullet head, height 900 µm, diameter 350 µm). The molds were centrifuged at 4000 rpm for 20 min. The excess enzyme solution was recovered by scraping, and the molds were dried at 25 °C for 30 min. Then the CUR-NCs suspension was added to the mold, and the foregoing operations were repeated three times. After that, the blank solution (30% PVP K30 (*w*/*v*)) was added, and the previous procedure was repeated once. Finally, the backing layer gels (20% PVA (*w*/*v*) and 20% PVP K30 (*w*/*v*)) were added to the mold, centrifuged at 2000 rpm for 3 min, dried at 25 °C for 12 h, and removed. MN patches with different enzyme concentrations and different morphologies were manufactured by the same manufacturing process, achieved by changing the enzyme solution concentrations and molds.

### 2.5. CUR-MNs Backing Layer Screening

By changing the materials and proportions to select the appropriate backing layer, the CUR-MNs with different backing layers were prepared by the aforementioned double-layer casting method. By comparing the properties of the lacking layer, CUR-NCs diffusion conditions and MNs skin insert rates, the optimal prescription of the backing layer was determined.

### 2.6. Morphology and Structure of CUR-MNs

The morphology and structure of the CUR-MNs were observed by Axio Observer inverted fluorescence microscope (Carl Zeiss AG, Oberkochen, Germany), stereomicroscope (SRZ-7045DM, KeXin, China), and Nova Nano SEM (FEI, Hillsboro, OR, USA).

### 2.7. Evaluation of Mechanical Properties of CUR-MNs

Bama miniature pig skin was purchased from Jingde Agricultural Products Sales Co., Ltd. (Hebei, China), which was frozen at −20 °C for further use. A certain size of pig skin was taken and soaked in normal saline for 1 h. The hair was carefully removed with a scraper, and the MNs were then inserted into the pig skin by pressing the thumb for about 30 s. After removing the MNs patch, the skin was stained with 0.5% aqueous solution of Trypan blue and observed by a stereomicroscope to calculate the skin insert rate.

As reported in our previous study [45], the mechanical force of MNs was tested by a TMS-pilot texture analyzer (FTC, Vienna, VA, USA). Firstly, the MNs were placed in a pin-up configuration. When the sensor first touched the tip of the MNs, the displacement and force test started. Then the sensor continued to probe. Lastly, when the sensor moved down 0.8 mm from the tip of MNs, the detection ended. 

### 2.8. Measurement of MNs Drug Loading

MNs tips with 30 intact were scraped from the patch with a blade and dissolved in 0.5 mL 50% (*v*/*v*) anhydrous ethanol solution. The CUR-NCs loading in MNs were determined by high-performance liquid chromatography (HPLC).

### 2.9. In Vitro Percutaneous Penetration Test

Franz diffusion cell experiment, with a vertical diffusion area of 2.25 cm^2^, was used to study the transdermal permeability of CUR-MNs in vitro. Pig skin slightly larger than the diffusion area was taken, soaked in physiological saline for 1 h, and pricked with MNs. The skin was superglued between the donor and recipient by cyanoacrylate to avoid skin displacement. In each diffusion tank, 5 g of weight was placed above the MNs to maintain pressure. 20% (*v*/*v*) PEG 400 aqueous solution was used as receptor medium, the experimental temperature was 37 ± 1 °C, and the rotational speed was 100 rpm. At 2, 4, 6, 8, 10, 12, and 24 h, the recipient culture medium was collected and supplemented by 1 mL fresh medium. The collected samples were centrifuged at 12,000 rpm for 10 min, and the supernatant was added to a shaded 96-well plate. To explore permeation circumstance, the fluorescence intensity of CUR-NCs was measured by a multifunctional microplate reader. In addition, the skin was removed and processed for the experiment on skin retention. The area with CUR diffusion was cut out, dissolved in 0.5 mL acetonitrile solution, ground with grinding beads, ultrasonic crushed (ultrasonic conditions: 3 s on, 2 s off, 5 min, 400 w power), and centrifuged at 12,000 rpm for 10 min. The supernatant was removed, and the skin retention of CUR was determined by HPLC.

### 2.10. In Vitro Skin Diffusion

The pig skin was soaked in normal saline for 1 h, and the hair was carefully scraped with a spatula. Then the CUR-MNs patch containing different concentrations of HAase was inserted and incubated at 37 °C for 30 min [46]. Meanwhile, the absorbent cotton moistened with normal saline was used to keep the skin moist and prevent the skin from becoming dry. The MNs were taken down, and the skin surface was wiped with normal saline. The orientation of the hair follicles was fixed with a pin so that the skin specimens were embedded in a tissue-freezing medium (O.C.T. Compound Leica, Mainz, Germany). Then the frozen skin was cut into sections (40 μm) with a freezing microtomy (Leica CM1950, Mainz, Germany) for visualization of skin insertion, observed and photographed under an inverted fluorescence microscope (Carl Zeiss AG, Oberkochen, Germany). According to previous studies, the diffusion area of CUR-NCs in the skin was measured by the software Image J 1.8.0 [47].

### 2.11. HPLC Conditions

The CUR was tested at 425 nm by an HPLC system (Agilent 1100, Santa Clara, CA, USA). On an XB-C18 column (Ultimate^®^; 250 × 4.6 mm, 5 µm) using a mobile phase of methanol and 3.6% (*v*/*v*) glacial acetic acid solution (75/25, *v*/*v*) at a flow rate of 1 mL/min [48,49].

## 3. Results

### 3.1. Fabrication of CUR-NCs

**Figure 1:**
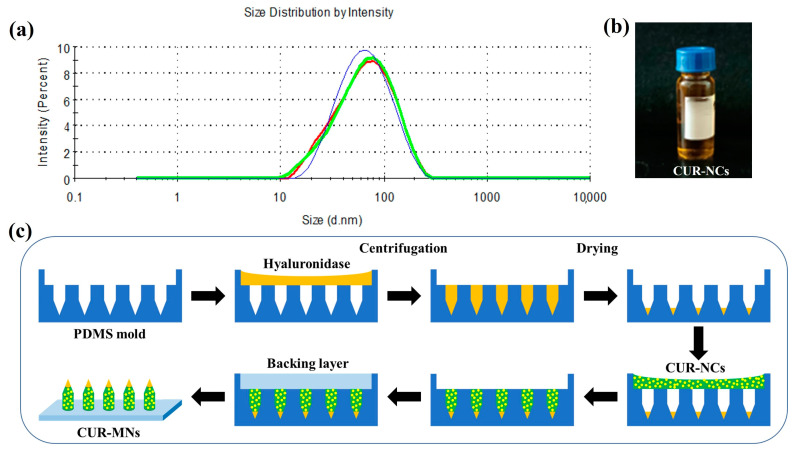
(**a**) The particle-size distribution by intensity of CUR-NCs (The blue, green, and red color curves represent the results of three tests of the same sample, respectively); (**b**) The nanosuspension of 55 nm-size CUR-NCs; (**c**) Fabrication process of CUR-MNs.

As shown in Figure 1a, the particle size of CUR-NCs was 54.73 ± 0.16 nm, and the PDI was 0.264 ± 0.003. The suspension of 55 nm-size CUR-NCs is displayed in Figure 1b, which is a suspension of light orange. By the double-layer casting method (Figure 1c), three different shapes of CUR-MNs with HAase in their tips were prepared. According to the scanning electron microscope images (SEM) and differential scanning calorimetry curves (DSC) produced in the previous study [36], 55 nm-size CUR-NCs are amorphous and spherical.

### 3.2. Morphology and Structure of CUR-MNs

**Figure 2:**
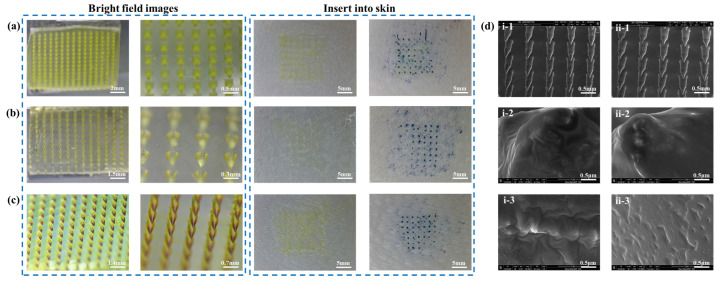
Bright field images of different shapes of CUR-MNs and skin insert rates under stereo microscope, (**a**) quadrangular cone, (**b**) cone, (**c**) bullet head; SEM images of (**d**) CUR-MNs (**i-1**–**i-3**) and CUR-MNs with HAase on the tip (**ii-1**–**ii-3**).

The overall and local bright-field images and skin insert rates of the three different shapes of MNs are displayed in Figure 2. All three MNs patches were 15 × 15 matrix arrays with the following shapes and skin insert rates: ① quadrangular cone, with a skin insert rate of 82% in Figure 2a; ② cone with a skin insert rate of 86% in Figure 2b; ③ bullet head with a skin insert rate of 92% in Figure 2c. Through comparative analysis, the bullet head showed a better skin insert rate. The enlarged SEM images of the CUR-MNs with HAase and without HAase are shown in Figure 2d, where the bullet head matrix array was clearly displayed.

### 3.3. Backing Layer Formulation Screening without Diffusion of CUR

**Figure 3:**
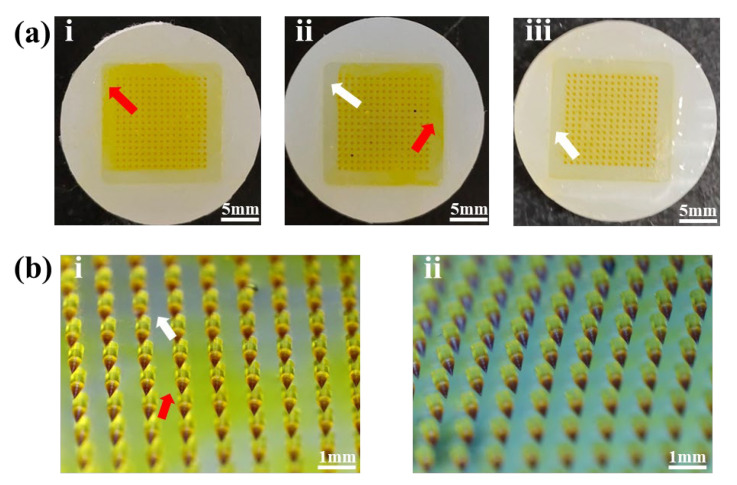
(**a**) Diffusion of the backing layers of different materials ((**i**) 20% PVA + 30% PVP K30; (**ii**) 30% PVA + 20% PVP K30; (**iii**) 20% PVA + 20% PVP K30 (*w*/*v*)); (**b**) CUR-NCs diffusion (**i**) and without diffusion (**ii**) in backing layers (red arrow—diffusion, white arrow—without diffusion).

In the preparation of the CUR-MNs, the interactions between the backing layer and HAase or CUR-NCs may occur, causing the CUR-NCs to diffuse from the needle tip into the backing layer. Therefore, in order to avoid the diffusion of CUR-NCs, the backing layer materials were changed to PVA and PVP K30, and the ratio of the two materials was adjusted. As shown in Figure 3(ai,ii), the diffusion phenomenon of CUR-NCs still existed. However, when backing layer materials were modulated to 20% PVA (*w*/*v*) and 20% PVP K30 (*w*/*v*), the diffusion of CUR-NCs was significantly decreased (Figure 3(aiii)). The non-uniformity of MNs is shown in Figure 3(bi), with the colors of the backing layer varied in shades, and the white arrows indicated areas where the CUR-NCs were without spread. However, as shown by the red arrows, it was clearly displayed that the CUR-NCs spread into the backing layer, thereby affecting the properties of the MNs. Presented in Figure 3(bii) were the MNs, which showed no obvious diffusion of CUR-NCs in the backing layer and a light yellow and uniform color. At the same time, the materials and skin insert rates (Table 1) of CUR-MNs with different backing layer materials showed a great insertion performance, with the skin insert rate over 85%. Based on the above results, 20% PVA (*w*/*v*) and 20% PVP K30 (*w*/*v*) may produce suitable softness and insertion performance backing layer.

### 3.4. Mechanical and Insertion Properties of CUR-MNs

**Figure 4:**
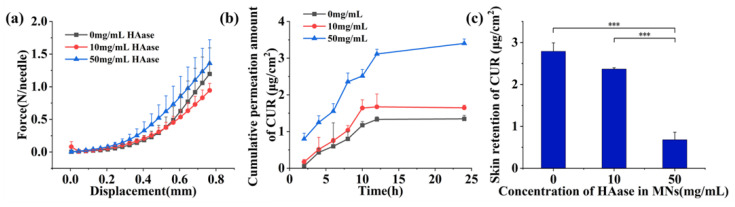
(**a**) Force–displacement curves, (**b**) Cumulative permeation and (**c**) Skin retention of CUR in MNs with different HAase concentrations. (Significance is denoted in the figure as *** *p <* 0.001).

The force–displacement curves of the CUR-MNs are shown in Figure 4a, indicating the hardness of the CUR-MNs with different concentrations of HAase. Three CUR-MNs could withstand a compressive force of ≥0.14 N/needle with good puncture ability [17,34]. Micro-pores were formed on the skin surface, and the skin insert rate of the 8 × 8 MNs matrix was 58/64 × 100% = 90.6% > 85% with sufficient insertion performance.

### 3.5. In Vitro Percutaneous Penetration

The results of in vitro transdermal cumulative permeation assay and skin retention of the CUR-MNs within 24 h were shown in Figure 4b and Figure 4c, respectively. The skin retentions of CUR with different HAase concentrations were also presented in Table 2. Compared to our previous studies, the transdermal permeation of the CUR-MNs increased while the skin retention decreased [13]. The permeation rate of CUR-NCs was faster for the first 12 h and slowed down for 12–24 h. In addition, the cumulative drug release in the 10 and 50 mg/mL groups was approximately 1.2 and 2.5 times of that in the 0 mg/mL group, respectively. There was no significant difference between the 0 and 10 mg/mL groups, which may cause by a low concentration of HAase. The HA may not be depolymerized completely at a low concentration. Therefore, the ECM may not be destroyed with slightly pro-osmotic ability. In addition, the pro-permeation effect of Haase at 50 mg/mL was greatly enhanced, and the skin retention was about 1/4 that of the 0 mg/mL group.

The experimental results showed that the HAase concentration of 50 mg/mL enhanced the transdermal permeation of CUR-NCs, with concentration-dependent behavior. The medium concentration of HAase would be added to verify the trend of promoting the permeability effect. The pro-osmotic capacity of HAase significantly improved the transdermal drug delivery efficiency, which may be attributed to depolymerizing HA by HAase. Thereby, the subcutaneous space was loose, and the ECM barrier was broken; thereafter, drug diffusion and penetration were promoted with a larger corona and longer distance [30].

### 3.6. In Vitro Skin Diffusion

**Figure 5:**
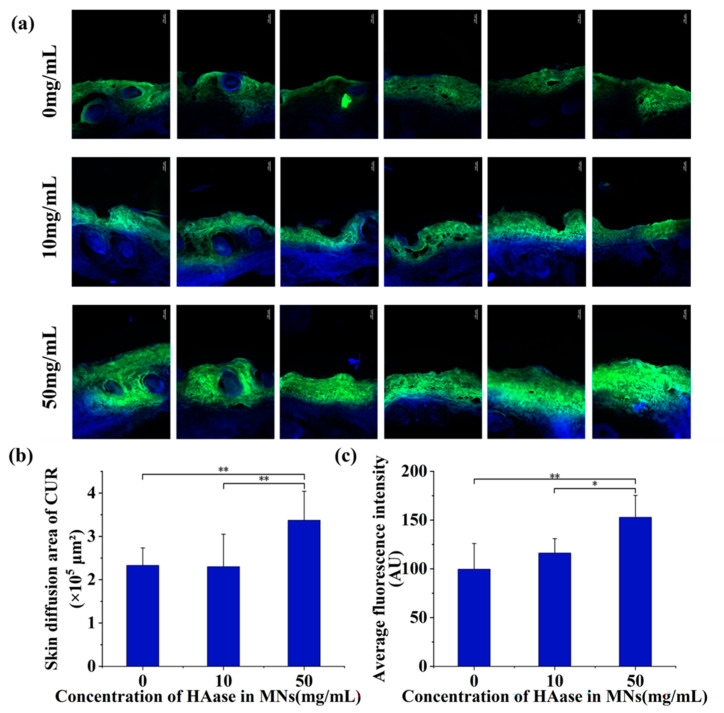
(**a**) Skin diffusion images of CUR-NCs (bar: 100 μm), (**b**) skin diffusion area and (**c**) fluorescence intensity of the CUR-MNs containing different HAase concentrations. (Significance is denoted in the figure as * *p <* 0.05, and ** *p <* 0.01).

As shown in Figure 5b and Table 3, HAase with different concentrations displayed different effects on the diffusion of CUR-NCs. The HAase with 50 mg/mL greatly promoted the transdermal absorption of CUR-NCs, while there was no significant difference in skin diffusion area between 0 and 10 mg/mL concentration groups. Fluorescence was observed in the SC of the skin, while the fluorescence area expanded and deepened into the active epidermis in the 50 mg/mL group. This result is consistent with the aforementioned in vitro transdermal permeation results, further demonstrating that 50 mg/mL HAase could significantly promote the transdermal absorption of CUR-NCs. In addition, the average fluorescence intensity of the slices was analyzed by Image J as well. As the data shown in Figure 5c and Table 4, the mean fluorescence intensity increased with increasing HAase concentrations, showing a certain concentration-dependent effect.

Combining the diffusion area and fluorescence intensity data, it can be reasonably inferred that the amount of CUR-NCs released from MNs increases with the increase of HAase concentration. However, there was no significant difference between the 0 and 10 mg/mL groups, possibly due to the low concentration of HAase without depolymerizing the HA to loosen the structure of ECM.

### 3.7. HAase-Based Diffusion Mechanism of CUR-NCs in Transdermal Delivery

**Figure 6:**
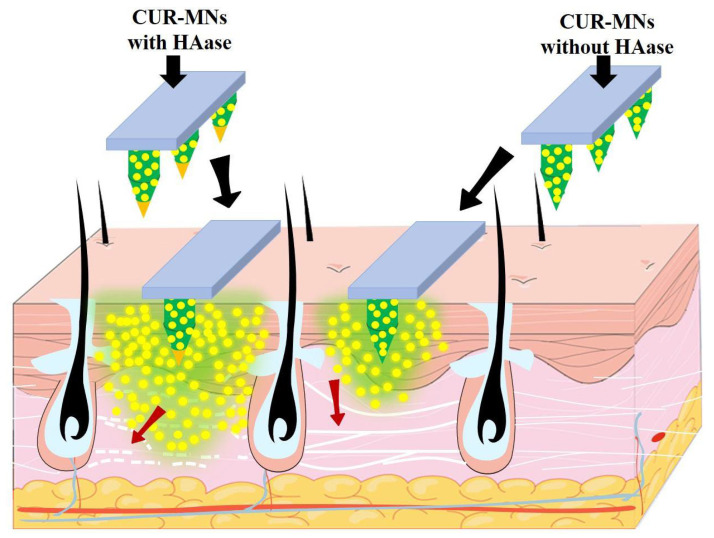
Mechanism of HAase enhancing intradermal diffusion of small-size CUR-NCs.

The CUR-MNs containing HAase were inserted into the skin; firstly, the HAase depolymerized the HA and loosened the ECM barrier. The small-size CUR-NCs, therefore, diffused deeper with a larger diffusion area than the CUR-MNs without HAase, showing better promotion and transdermal absorption of CUR (as shown by the red arrows in Figure 6). This mechanism is also consistent with the experimental data of in vitro penetration and diffusion. For the 50 mg/mL group, more cumulative permeation and less skin retention were demonstrated, along with an increase in skin diffusion area. In the 10 mg/mL group, the effect of MNs containing HAase on promoting skin penetration and diffusion was significantly weakened, which might be due to the lower HAase concentration. 

### 3.8. Drug Loading of CUR-MNs

The drug loading of CUR-MNs was 0.37 ± 0.02 μg/needle by measuring the samples according to the HPLC conditions corresponding to Section 2.7.

## 4. Conclusions

In this study, 55 nm-size CUR-NCs were fabricated and concentrated as a CUR-NCs suspension. MNs with or without HAase in the tip were prepared by a double-layer casting method, with a drug loading of 0.37 ± 0.02 μg/needle. CUR-NCs in MNs with HAase showed higher penetration and lower skin retention within 24 h. In addition, CUR-MNs containing HAase were also confirmed to have a larger transdermal diffusion corona area and deeper penetration distance. This excellent increased diffusion phenomenon may be caused by the insertion of MNs, which loosens the SC barrier to deliver small-size CUR-NCs to the dermal firstly. The second improvable strategy might be the ability of HAase to react specifically with HA in the ECM. HA was degraded, and the ECM was loosened, creating promising conditions for the increased diffusion and absorption of CUR-NCs. The combination of MNs and HAase may provide a better effect on improving the efficiency of transdermal drug delivery of insoluble drugs.

Thus, HAase-based MNs could promote the transdermal penetration efficiency of CUR-NCs, and the behavior showed some concentration dependence. By controlling the concentration of HAase and the materials’ proportion of MNs matrix, it is expected to achieve efficient transdermal delivery of insoluble drugs. The combination strategy of HAase, MNs and NCs might offer greater hope for the improvement of clinical skin disease problems with poorly soluble drugs.

## Figures and Tables

**Table 1:** Different backing materials and the skin insert rates of MNs.

Materials of Backing Layer		Skin Insert Rate (%)
PVA (%, *w*/*v*)	PVP K30 (%, *w*/*v*)	
15	35	92
20	20	91
20	30	83
25	25	82
30	20	85

**Table 2:** Cumulative permeation amount of CUR in MNs.

Concentration of HAase in MNs (mg/mL)	Skin Retention of CUR (μg/cm^2^)	STDEV.P
0	2.79	0.20
10	2.37	0.03
50	0.68	0.18

**Table 3:** The skin diffusion area of the CUR-MNs containing different HAase concentrations.

Concentration of HAase (mg/mL)	Skin Diffusion Area of CUR (×10^5^ μm^2^)	STDEV.P
0	2.33	0.40
10	2.30	0.75
50	3.37	0.67

**Table 4:** The fluorescence intensity of the CUR-MNs containing different HAase concentrations.

Concentration of HAase (mg/mL)	Average Fluorescence Intensity (AU)	STDEV.P
0	99.65	26.36
10	116.36	14.62
50	152.97	22.41

## Data Availability

Not applicable.
